# A Porous Nanostructured ZnO Layer for Ultraviolet Sensing with Quartz Crystal Microbalance Technique

**DOI:** 10.3390/mi14081584

**Published:** 2023-08-11

**Authors:** Abil S. Asvarov, Arsen E. Muslimov, Soslan S. Makhmudov, Vladimir M. Kanevsky

**Affiliations:** 1 Shubnikov Institute of Crystallography, Federal Scientific Research Center “Crystallography and Photonics”, Russian Academy of Sciences, Leninsky Prospect, 59, 119333 Moscow, Russia; amuslimov@mail.ru (A.E.M.); kanev@crys.ras.ru (V.M.K.); 2 Institute of Physics, Dagestan Federal Research Center, Russian Academy Sciences, Yaragskogo Str., 94, 367015 Makhachkala, Russia; Draiw76@mail.ru

**Keywords:** porous structure, ZnO, sputtering, thermal oxidation, QCM, UV sensitivity

## Abstract

Porous films of metals and metal oxides have gained growing attention as potential materials for use in applications that require large, specific surface areas, such as sensors, supercapacitors, and batteries. In this study, a “black-metal”-like porous Zn–ZnO composite layer was grown by room temperature co-sputtering of Zn metal and ZnO:Ga (3 at/%) ceramic targets. Following deposition, a porous ZnO layer was obtained by a subsequent thermal annealing process at 400 °C in air. The morphology and structural properties of the obtained porous layered objects were analyzed. The porosity and chemical characteristics of the nanostructured ZnO layer obtained with the method herein described make it suitable to be used as a sensitivity-enhancing active layered element in quartz crystal microbalance (QCM)-based ultraviolet (UV) sensors. The prepared resonant ZnO/QCM sensors under UV radiation exhibited maximum shift up to 35 Hz for several “on-off” UV cycles, excellent response, and recovery times of 11 and 12 s, respectively.

## 1. Introduction

Porous layers of both metals and their oxides are the objects of many studies due to the fact that the porosity often opens up a plethora of new application possibilities by virtue of the increased surface area [1,2,3,4].

Highly porous metal layers, the so-called black metals (BM), having a moth-eye-like surface morphology, exhibit high absorption in a wide range of the electromagnetic spectrum [5,6,7]. BMs have found applications in such areas as solar energy absorbers, radiative heat exchangers, camouflage, electrodes of photovoltaic cells, and biosensors [8,9,10,11]. 

In turn, embedding porosity into metal oxides can also give them new properties and advanced performance in many applications. Particularly, porous metal oxides of transition metals have been of interest for catalytic applications as they generally have multiple valence states and can readily form a redox cycle between the high and low oxidation states [12,13], while porous materials based on two-dimensional transition metal carbides and nitrides show huge potential in the development of ultra-sensitive flexible pressure sensors for intelligent health monitoring [14,15], as well as semiconducting metal oxides are already finding increasing use in application fields such as gas- and bio- sensing, electromagnetic radiation photodetection, self-cleaning, hydro-, ice-, oleo- phobic, anti-reflective, anti-bacterial and other functional “smart” coatings [16,17,18,19,20,21,22,23].

Regarding metal oxide semiconductors, inexpensive zinc oxide (ZnO) is attracting inextinguishable interest since it has a unique combination of electrophysical, optical, and surface chemical properties, and routes to achieve various types of porosity in it are already well developed using manifold methods (PVD, CVD, wet chemical synthesis, solid-state synthesis, etc.) [23]. Although wet chemical synthesis of various porous ZnO structures is regarded as one of the more efficient and easier low-temperature methods for large-scale applications, the precious controlling architecture of ZnO nanostructures is still challenging [19]. Despite the fact that each of the above-mentioned methods has certain limits in either large-scale applications or substrate selection due to their obstacles such as high temperatures, expensive equipment, complexity, harvesting, and toxicity, in our opinion, a two-step synthetic approach, involving magnetron sputtering of Zn BM layers and their oxidation by short-term thermal annealing in ambient air at relatively low temperatures, is the least expensive and the simplest for synthesizing layered ZnO with the desired porous nanocrystalline structure [24]. Importantly, both steps of this approach are well compatible with standard semiconductor manufacturing processes. Mechanisms for the magnetron sputtering deposition of porous Zn BM layers, taking into account the influence of the substrate temperature, gas-phase clustering of sputtered particles, pressure, and composition of the working gas, have been proposed by several research groups [25,26,27]. In addition, it has been demonstrated that using very mild conditions for the thermal annealing step in terms of its duration and temperature allows the formation of ZnO layers, the porosity of which is close to that of the originally deposited Zn BM layers [25,27,28].

Li L. et al. have pointed out that the incorporation of oxygen during sputtering resulted in the formation of two phases, Zn and ZnO, and the promotion of a porous cauliflower-like structure made of interconnected fine particles, eventually showing a Zn/ZnO core-shell configuration [24]. In turn, Park S.Y. et al. indicate that porous Zn–ZnO composite films deposited from Zn targets in the presence of a small amount of oxygen exhibit improved adhesion properties and superior reliability while maintaining a high level of porosity [27]. From the results presented in [26], it can also be assumed that the residual gases in the vacuum chamber could contribute to the formation of amorphous ultraporous Zn BM layers, since on the substrate, cooled by liquid nitrogen vapor, in addition to zinc clusters and atoms, molecules of residual gases could also be effectively adsorbed. From the above, it can be assumed that the formation of the porous composite Zn–ZnO layer at the first step could promote the earlier nucleation process of oxides in the subsequent oxidation step, inhibit evaporation of molten components, and limit preferential growth of ZnO along specific directions, thus resulting in the formation of porous nanocrystalline ZnO films with a porous structure close to that observed for the original composite Zn–ZnO layer. 

Thus, we herein report the use of a modified two-step approach for forming porous ZnO layers, wherein a process of co-sputtering Zn and Ga-doped ZnO targets is employed to fabricate the porous Zn–ZnO layer at the first stage, with its subsequent oxidation to ZnO at the second thermal annealing stage. Finally, the porous ZnO layer was fabricated on the surface of the 5 MHz AT-cut α-quartz crystal in order to test the possibility of creating a resonant type ultraviolet (UV) radiation sensor, in which the operation principle is based on detecting a change in the frequency of the oscillator due to the desorption of oxygen from the surface of the layered ZnO upon UV irradiation [29,30,31].

## 2. Materials and Methods

### 2.1. Synthesis and Characterization of Porous Zn–ZnO and ZnO Layers

Porous Zn–ZnO composite layers were deposited using magnetron co-sputtering of Zn metal and ZnO:Ga ceramic targets in pure Ar gas medium at a working pressure of 2 Pa. The Zn target 75 mm in diameter with a purity of 4N was sputtered in mid-frequency mode with a power of 200 W, while the ceramic target made of ZnO doped with Ga (3 at.%) 75 mm in diameter was sputtered in radio-frequency mode with a power of 50 W. The porous composite layer was deposited on a substrate fixed on an unheated disk holder rotating around its axis at a distance of 15 cm from the faces of magnetron units. Thus, during the 10 min deposition, the substrate alternately found itself either opposite the Zn sputtering zone or opposite the ZnO:Ga sputtering zone. 

After the deposition by co-sputtering, to form the fully oxidized ZnO layer, the composite layer was subjected to post-oxidation using a tube furnace (Nabertherm, Lilienthal, Germany) under an air atmosphere at temperatures up to 400 °C.

The surface morphology, phase, and chemical composition, as well as the microstructure of the as-deposited and annealed porous layered samples, were investigated using a standard set of characterization techniques: scanning electron microscopy (FIB/SEM Quanta 200 3D, FEI, Hillsboro, OR, USA), energy dispersive X-ray spectroscopy (INCA Energy 250, Oxford, UK), and X-ray diffraction (X’PERT PRO MPD, Malvern Panalytical B.V., Almelo, The Netherlands). The SEM study of the sample morphology was carried out at an accelerating voltage of 2 kV and an electron probe current of 20 pA, while the EDX spectra were recorded in the mode with an accelerating voltage of 20 kV. The XRD spectra were obtained in the Bragg–Brentano geometry with a step size of 0.01° using a CuKα-radiation source operated at 40 kV and 30 mA.

### 2.2. Fabrication of the ZnO/QCM-Based UV-Sensitive Structure and Investigation of Its UV Sensor Characteristics

In conformity with the results of the optimization of the two-stage technique, it turned out to be possible to form a resonance-type UV sensor structure using α-quartz as a resonator since the maximum temperature of the process (at the second post-oxidation stage) of the formation of the porous ZnO layer was lower than the phase transition temperature in quartz. Accordingly, a 1 inch, 5 MHz, AT-cut standard α-quartz crystal with patterned Au electrodes (Stanford Research System, Sunnyvale, CA, USA) was used to form the resonant ZnO/QCM structure for UV sensing. 

To investigate the UV radiation sensitivity, the porous ZnO/QCM structure was irradiated with a UVC lamp DKB-7 (NIIIC Lodigina, Saransk, Russia) emitting in the wavelength range 200—280 nm (the dominant emission wavelength of 253.7 nm) with a UVC radiation intensity of ≥80 μW/cm^2^ at a distance of 15 cm. In this case, the registration of changes in the resonant frequency of the ZnO/QCM structure with the UV lamp turned “ON” or “OFF” was carried out in the relative frequency mode with a gate time of 1 s and resolution of 0.1 Hz using a digital controller model QCM200 (Stanford Research Systems, Sunnyvale, CA, USA) equipped with an appropriate crystal holder and a 5 MHz crystal oscillator, QCM25. 

## 3. Results and Discussion

### 3.1. Two-Stage Technique for for Forming Porous ZnO Layers

When realizing a sensing layer/QCM-based sensor, a trade-off must be found: high porosity of the sensitive layer means a larger adsorbing area and thus a larger sensitivity; nevertheless, too high porosity of the sensitive layer usually leads to its weak adhesion to the substrate, which, if QCM plate is used as a substrate, may ultimately affect the stability and reliability of resonant “sensitive layer/QSM”-based devices due to destruction and delaminating of the sensitive layer [27,32,33]. 

For the case of the deposition of zinc-containing porous layers, it has been confirmed by various groups [23,24,25] that the pure porous Zn film fabricated by injecting only inert Ar gas into the system exhibited a high porosity with BM morphology but showed poor substrate adhesion and low mechanical reliability. At the same time, it is already known that adhesion can be improved by a small addition of oxygen to inert argon [27,34]. 

To get away from the reactive sputtering of the Zn target in an Ar–O_2_ gas mix, we used the technique of co-sputtering of Zn metal and ZnO-based ceramic targets in a pure Ar medium to deposit porous Zn–ZnO composite layers. In this case, the sputtered ceramic target supplies oxygen spices, and the amount of oxygen can be effectively controlled by the ratio of the powers supplied to the magnetrons.

**Figure 1:**
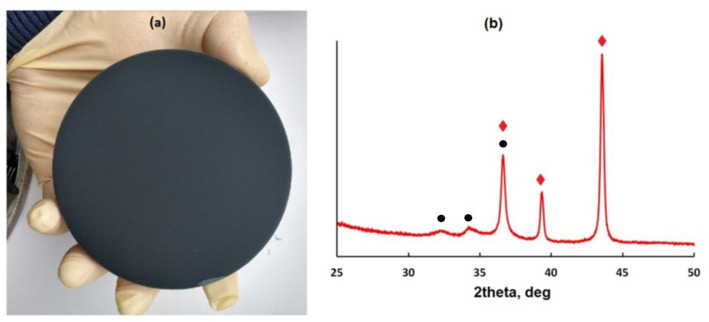
The appearance (**a**) and XRD pattern (**b**) of the as-deposited Zn–ZnO layer. In the pattern, the peaks corresponding to the Zn metal phase and the ZnO phase are marked with red diamonds and black circles, respectively.

Figure 1a shows the appearance of the as-deposited ~1 µm thick Zn–ZnO composite layer. Its surface appears matt black, which is a confirmation of a highly developed (grainy or porous) surface [7,20,22]. XRD data reveal the presence of two phases in the deposited composite film. The XRD pattern (Figure 1b) is composed of the strongest peaks related to Zn (002), (100), and (101) crystallographic planes (JCPDS ref. number Zn 87-0713) and weaker peaks related to ZnO (100) and ZnO (002) planes (JCPDS ref. number ZnO 89-1397). Taking into consideration the relative peak intensities for the observed hexagonal Zn phase, there is no preferential orientation in any direction for the Zn metal crystallites. The broadness is noticeably lower for the Zn peaks than for the ZnO peaks, thus meaning that the Zn phase has larger crystalline domains. In general, the dominance of the Zn phase over the ZnO phase observed in the XRD pattern (regarding both the intensity and the width of the peaks) of the composite Zn–ZnO layer is in agreement with the technological conditions of its deposition, under which the intense sputtering of the zinc target (200 W) was actually assisted by the less intense sputtering of the ceramic target (50 W).

**Figure 2:**
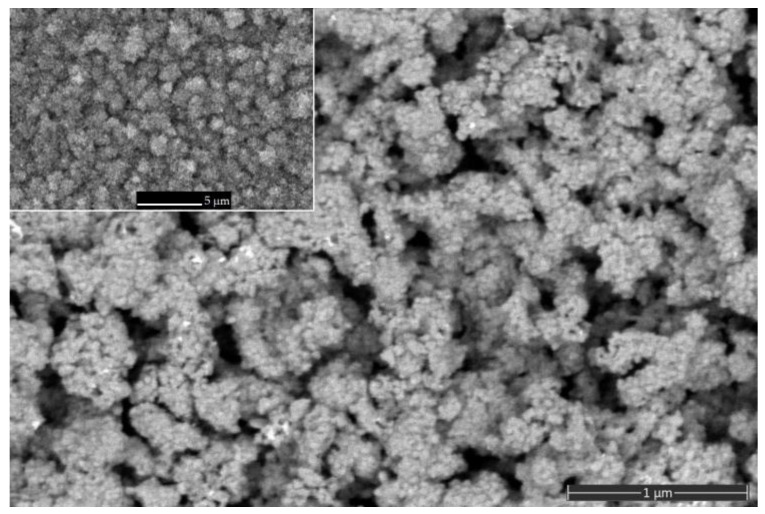
High-magnification and low-magnification (inset) SEM micrographs showing the top views of the as-deposited Zn–ZnO composite layer.

The top surface of the as-deposited Zn–ZnO composite layer is shown in Figure 2 at low and high magnifications. From the respective images, it is evident that for the co-sputtering case, the porous nanostructured morphology was obtained containing the well-recognized micro-sized agglomerates, having a shape similar to cauliflower appearance, which in turn consist of densely packaged nanograins having an average size in the range of 20–60 nm. This morphology is responsible for the BM-like behavior of the Zn–ZnO composite layer.

**Figure 3:**
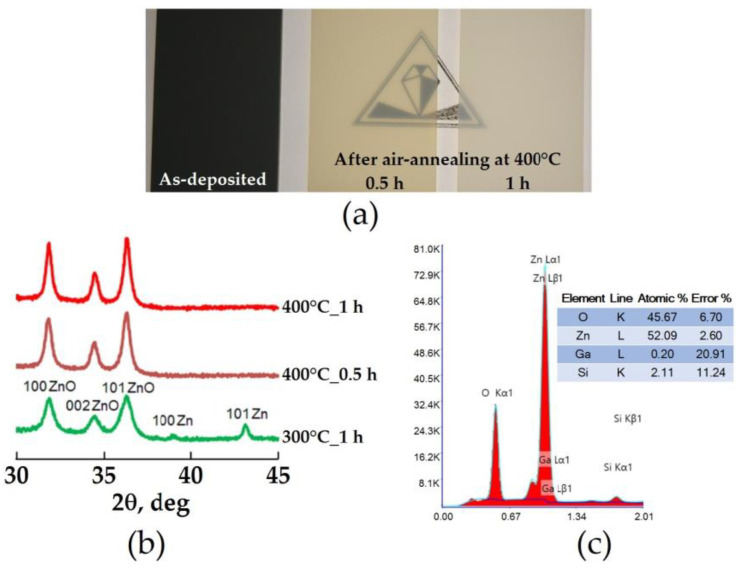
(**a**)—the appearance of the Zn–ZnO composite layer on glass before (left) and after air-annealing at 400 °C for 30 min (in center) and 1 h (right); (**b**)—XRD patterns of the Zn–ZnO composite layer after air-annealing at 300 °C for 1 h and 400 °C for 30 min and 1 h; (**c**)—EDX data for the Zn–ZnO composite layer after air-annealing at 400 °C for 1 h.

Further, the transformation of the layered Zn–ZnO composite into a pure oxide state under the thermal oxidation treatments was studied (Figure 3). Visual inspection showed that the coloration of the deposited composite changed after the air-annealing treatment, from a matt black through matte yellow to a matt milky appearance. The yellowness of the layer decreased with an increase in both the annealing temperature from 300 to 400 °C and the duration of the thermal treatment (Figure 3a). According to XRD data, the observed color transformation is due to the oxidation of the Zn metal phase (Figure 3b). As a result of hourly annealing at 300 °C, the two-phase state is preserved in the composite layer. In the corresponding XRD pattern, in addition to the ZnO peaks, there are still minor peaks corresponding to the metal phase, while an increase in the annealing temperature to 400 °C led to the complete disappearance of the Zn crystalline phase even at a 30 min duration of thermal treatment. When the duration of 400 °C annealing is increased to 1 h, only a slight narrowing of the ZnO peaks has a place to be, thus meaning that with an increase in the dose of thermal exposure, the average size of ZnO crystalline domains increases. The mean value of the ZnO crystallite size in this sample, determined using the Scherrer equation [35] with 101 ZnO peak data, is about 40 nm. 

Alivov et al. [36] inferred that Zn oxidation is a rapid process because the entire transformation of Zn thin film to ZnO depends not so much on the oxidation duration as on the oxidation temperature. They noticed that complete oxidation was achieved in the first 15 min. However, an increase in the oxidation duration improves the quality of emerging ZnO phase crystallinity. Analysis of data from other researchers shows that there was no unanimous temperature for complete oxidation. Many authors have observed the transition from the Zn thin film to the ZnO one during annealing at temperatures ranging from 300 to 700 °C [24,25,26,27,37,38,39]. Since oxidation is achieved by oxygen diffusion, the film thickness, structure, and composition might be the important parameters controlling the complete transition temperature. It is obvious that, if the film contains some porosity, the oxidant species may easily diffuse into an oxidizable substance, which consequently accelerates the oxidation process. Gazia et al. [25] reported that the total oxidation of Zn nanobranched layers is achieved at 380 °C for 60 min. In less porous Zn–ZnO layers, Park et al. [27] observed a complete transformation to ZnO during a very short 5 min annealing at 500 °C. In turn, Aida et al. [37] had observed the lowest complete oxidation temperature, which was equal to 300 °C. They attributed this to the presence of a significant amount of oxygen in the as-deposited Zn film with a dense and continuous microstructure, which is not the case in the other studies. Consequently, the oxygen presence in the as-grown film promotes ZnO nucleation at lower temperatures; therefore, the complete oxidation temperature at 400 °C in our Zn–ZnO layer observed from XRD results is well consistent with data from other research groups [25,37,39].

Mainly for a qualitative analysis and for the evaluation of the possible presence of impurities, the chemical composition of the sample annealed at 400 °C for 1 h was studied by means of EDX spectrometry, which confirmed the presence of the main Zn and O chemical elements, along with Ga and Si contributions, which can be attributed to doping and substrate signals, respectively. Although the EDX qualitative analysis showed some excess of Zn relative to oxygen in the sample annealed at 400 °C for 1 h (Figure 3c), annealing at higher temperatures was not carried out to prevent possible excessive recrystallization growth of ZnO crystallites and the associated deterioration in porosity. It is interesting to note that the Zn/Ga ratio in this annealed sample increased by a factor of ~8 compared to that in the sputtered ceramic target of ZnO with 3 at.% Ga. From this, we can conclude that the as-deposited Zn–ZnO composite layer consisted of seven parts of the metal phase and one part of the oxide phase.

**Figure 4:**
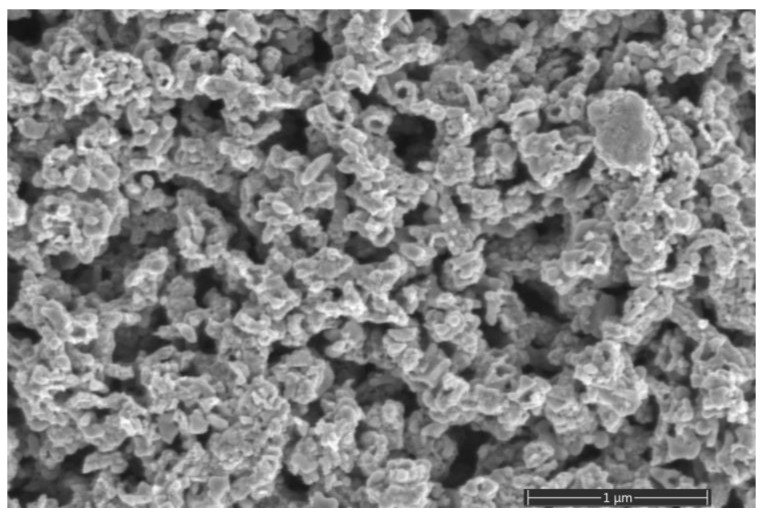
SEM micrograph showing the top views of the porous layer after air annealing at 400 °C for 1 h.

Figure 4 shows the surface morphology of the final porous ZnO layer prepared by annealing the Zn–ZnO composite layer at 400 °C for 1 h. A branched, porous nanostructure of the oxide layer is visible. From the comparison of the morphological characteristics between the as-deposited Zn–ZnO layer (Figure 2) and the annealed ZnO layer, some increase in the average size of aggregated nanograins forming a branched network is observed. However, as can be seen after annealing, the ZnO layer still exhibits an acceptable, high degree of porosity. Therefore, the annealing temperature of 400 °C and duration of 1 h were selected to prepare the “sensitive porous ZnO/QCM” structure by the two-stage technique. In addition, this value of the annealing temperature is noticeably lower than the irreversible phase transition temperature for α-quartz of 573 °C [29].

### 3.2. Fabrication of the “Porous ZnO/QCM”-Based Structure and Characterization of Its UV Sensitivity

**Figure 5:**
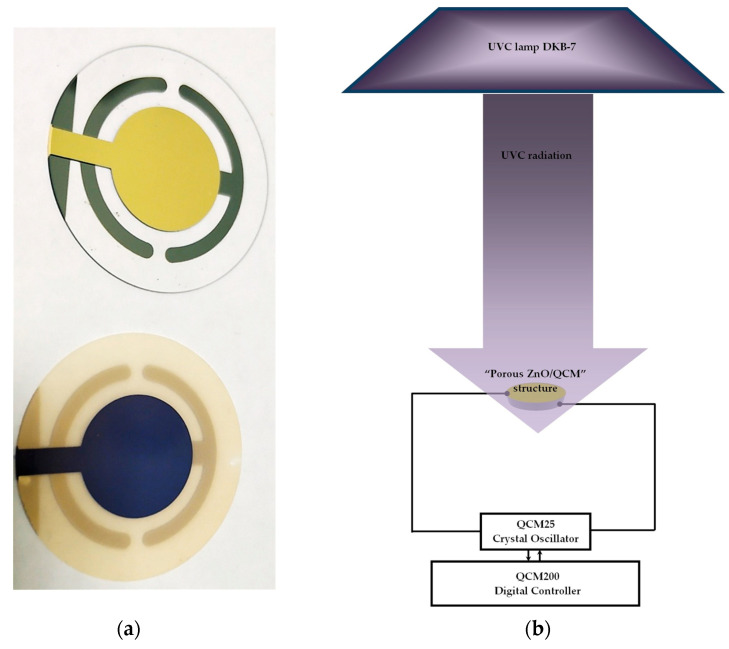
(**a**) the appearance of the bare QCM plate (**top**) and the QCM plate covered with ~1 µm thick, porous ZnO layer (**bottom**); (**b**) Configuration of UV radiation detection system.

Further, to form the “sensitive porous ZnO/QCM” structure, the ~1 µm thick, porous ZnO layers were deposited on the bare standard QCM plate with an Au electrode pattern from Stanford Research System (USA). Figure 5a shows a visualization of the bare QCM plate and prepared “porous ZnO/QCM” structure, while Figure 5b shows a scheme to investigate the response of the QCM coated with a porous ZnO layer to UV radiation.

The system consisted of a special holder with a “porous ZnO/QCM” structure connected to the QCM200 digital controller through the QCM25 crystal oscillator and the lamp DKB-7 as a UVC light source. The distance between the UVC source and the holder was 15 cm. The real-time frequency shift Δ*f* relative to the initial resonant frequency *f*_0_ of the “porous ZnO/QCM” structure was recorded according to Δ*f* = *f − f*_0_.

**Figure 6:**
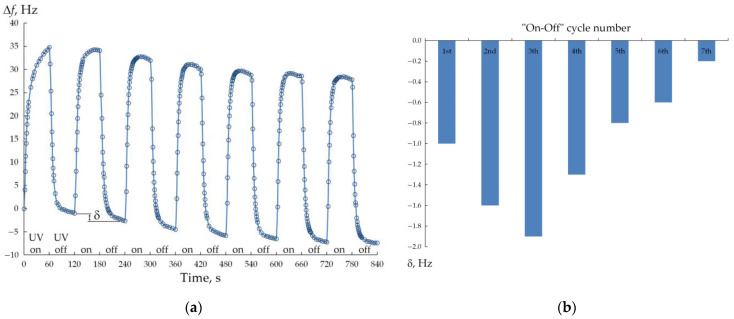
Response of the “porous ZnO/QCM” structure to periodically exposed UVC light (**a**) and values of drift δ observed within each of the successive “on-off” cycles (**b**).

The UVC source was turned “on” for 1 min, and the positive shift Δ*f* was registered in real time (accordingly, the frequency *f* increased relative to *f*_0_). Next, the source was turned “off” for 1 min, during which a reverse change in Δ*f* was observed. This two-minute “on-off” cycle was repeated eight more times to clarify the sensing repeatability of the “porous ZnO/QCM” structure. Figure 6a shows the shift Δ*f* of the time curves for the fabricated “porous ZnO/QCM” structure under the repeated UV on-off cycles. 

As shown in Figure 6a, the whole of the Δ*f* of the prepared structure could be reversibly modulated by UV irradiation by repeatedly switching the UV light on and off for the same time intervals of 60 s, but there are a few issues to point out. 

Firstly, the transient response for the first “on-off” cycle looks different compared to the following cycles. In the first cycle, the shift Δ*f* increases quickly at the moment of the UV light source turning on, and then it increases more and more slowly to form a tail rather than a saturation shelf. During subsequent “on-off” cycles, Δ*f* also increases rapidly at the beginning of the UV light source turning on and reaches a stable value quickly, and an obvious shelf appears.

Secondly, when the UV illumination is interrupted, the shift Δ*f* decreases quite quickly and, by 60 s, is below zero, i.e., there is some zero-level drift δ. Thus, the second “on-off” cycle started with a negative value of Δ*f*. Moreover, the drift δ is observed after the completion of each cycle relative to the value at the start of the cycle, and thus the cumulative drift δ_total_ after nine cycles was 7.4 Hz (Figure 6b). However, it should be noted that the magnitude of the change in the resonant frequency *f* within each “on-off” cycle did not change (~35 Hz) for all nine cycles performed.

It has been shown earlier [29,30,40] that the effect of applying an additional layer to the QCM can be described by including an additional parallel branch with capacitance *C*_ZnO_ in the Butterworth–van Dyke electrical model for a quartz crystal resonator [41]. In our case, the change in the resonant frequency *f* of “ZnO/QCM” structures under UV action is associated with a change in the parallel impedance of the Butterworth–van Dyke equivalent circuit, mainly due to a change in capacitance *C*_ZnO_. Thus, the sensing principle of the ZnO-QCM-based UV sensor is mainly explained by some change in the capacitance of the porous ZnO layer due to the generation of electron-hole pairs in it [30,40,42], and not by the principle of direct detection of a mass change by the QCM due to desorption from the ZnO surface of previously adsorbed spices (O_2_, OH, organic molecules, etc.) [31].

The special behavior in the transient response for the first “on-off” cycle observed in our experiment can be explained by the difference in environmental conditions in the room in which the second stage of manufacturing the porous structure was carried out from the conditions in the second room in which the sensitivity measurements were carried out. In the second room, measures were taken to maintain the relative humidity at a constant level (RH = 50%). So, we believe that the composition of spices adsorbed on the surface of porous zinc oxide before the first cycle could differ from the composition of spices newly adsorbed on zinc oxide during the first UV-off cycle (from 60 to 120 s). The difference in composition of adsorbed spices can affect the kinetics of the release of trapped spices from the ZnO surface due to the neutralization of their negative charge by photogenerated holes [31,42].

In turn, the drift δ observed in Figure 6a after each cycle was, in our opinion, due to temperature effects. According to the specification of the used QCM crystal, its intrinsic temperature coefficient is at least ~1 Hz/°C around room temperature. Unfortunately, during the experiment, the surface temperature of the “porous ZnO/QCM” structure was not controlled in a special way. It is quite possible that at the ambient temperature maintained in the room at 24 °C, the surface of the structure was nevertheless gradually heated by several °C during nine cycles under the action of parasitic infrared radiation from the UV lamp. In favor of this version, it can be seen from Figure 6b that the drift value recorded after each cycle during the first three cycles increased and then gradually decreased during subsequent cycles, which indicated that thermal equilibrium had been reached in the “weak IR source (UV lamp)—“porous ZnO/QCM” structure—room environment” system. In fact, the drift during the last 9th cycle was only 0.2 Hz.

**Figure 7:**
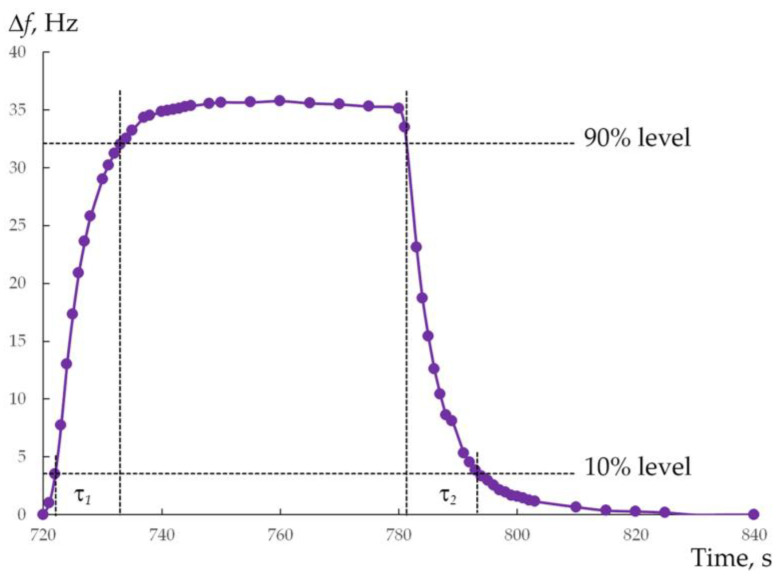
Frequency response curve of the “porous ZnO/QCM” structure during the 9th “on-off” cycle.

Figure 7 shows the transient response for the 9th “on-off” cycle, which has been corrected for the accumulated drift at the beginning of this cycle (−7.2 Hz) as well as the drift that occurs in the 9th cycle itself (−0.2 Hz). The correction procedure is presented in Appendix A. It can be seen that the frequency shift ∆*f* quickly reaches a stable value of ~35 Hz at the beginning of the UV light source turning on. This phenomenon can be explained by the fact that the process of the carriers’ generation is saturated quickly in the porous ZnO nanocrystalline layer [38]. From the curve in Figure 7, response time τ_1_, defined as the time taken for the shift in resonant frequency to change from 10% to 90%, is 11 s. 

At the same time, it can be seen that the structure is also characterized by a short recovery time τ_2_ = 12 s. The recorded response and recovery times for the “porous ZnO/QCM” structure are significantly better than most of the other reports [30,42,43,44,45]. Typically, ZnO-based UV-sensitive structures are characterized by a time-consuming recovery process, and typical recovery times in them have been reported to be in the range of 60 s and above. Qualitatively, the recombination of photon-generated carriers contains two different modes. One is the bulk recombination inside the ZnO, which is fast. The other is the surface recombination caused by the adsorbed oxygen at the surface of the ZnO, which is slow [42]. The recovery process is determined by the slow recombination mode. We believe that, in the case of our “porous ZnO/QCM” structure, the developed porous network in the nanostructured ZnO layer [29,30], as well as the fact of low-level doping of ZnO with Ga [46,47,48], contribute to the acceleration of gas adsorption by the surface in the absence of UV illumination.

Thus, the results of the study show that the addition of a sensitive porous ZnO layer on the surface of the QCM is a good option for a UV radiation sensor. However, the universal sensitivity of QCM to anything that can change its resonant frequency increases the chance of various interferences. To improve the UV-sensitive structure, it is imperative to find ways to further enhance the specificity of the developed porous ZnO/QCM structure to respond only to UV and not to other environmental conditions [40,49].

## Figures and Tables

**Figure A1:**
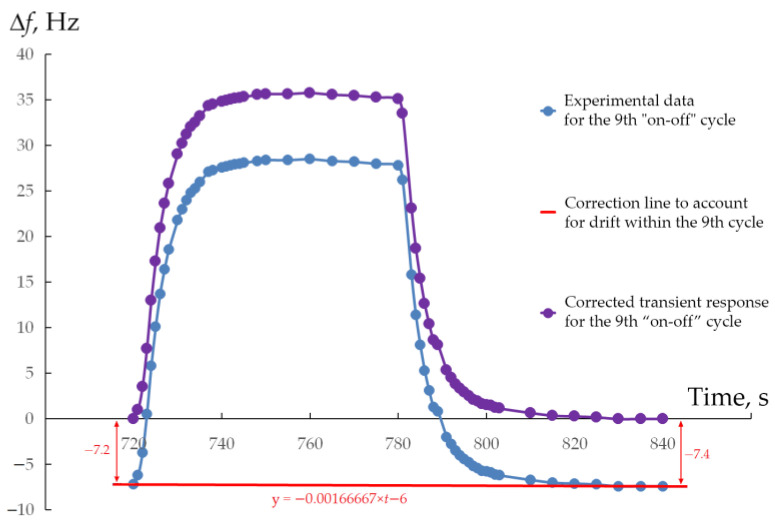
Experimental and corrected transient response curves of the “porous ZnO/QCM” structure for the 9th “on-off” cycle.

## Data Availability

Data sharing is not applicable to this article.

## Appendix A

Here, the corrected curve (purple) of the response was obtained by subtracting from the experimental curve (blue) the linear approximation of the drift contribution given by the function y = *a* × *t* + *b* (red), where *t* is the time, *a* = −0.00166667 is the coefficient taking into account the frequency drift δ = 0.2 Hz during the 9th cycle, and b = −6 is the coefficient that takes into account the accumulated drift of 7.2 Hz by the beginning of the 9th cycle.
